# *Rumex acetosa* modulates platelet function and inhibits thrombus formation in rats

**DOI:** 10.1186/s12906-020-02889-5

**Published:** 2020-03-23

**Authors:** Dahye Jeong, Muhammad Irfan, Dong-Ha Lee, Seung-Bok Hong, Jae-Wook Oh, Man Hee Rhee

**Affiliations:** 1 Laboratory of Veterinary Physiology and Cell Signaling, College of Veterinary Medicine, Kyungpook National University, Daegu, 41566 Republic of Korea; 2 Department of Biomedical Laboratory Science; and Molecular Diagnostics Research Institute, Namseoul University, Cheonan, 31020 Republic of Korea; 3 Department of Clinical Laboratoy Science, Chungbuk Health & Science University, Cheongju-si, Chungbuk 28150 Republic of Korea; 4 Department of Animal Biotechnology, Konkuk University, Seoul, 05029 South Korea

**Keywords:** Antiplatelet agent, Integrin α_IIb_β_3_, MAPK, Platelets, *Rumex acetosa*, Thrombosis

## Abstract

**Background:**

The *Rumex acetosa* has been used in medicinal treatment, food technology and phytotherapeutics in Eastern Asia and many other countries. However, its effect on cardiovascular system and antiplatelet activity remained to be known. In this study, we examined the antiplatelet activity of *R. acetosa* in detailed manner to understand underlying mechanism.

**Methods:**

To study this, whole blood was obtained from male Sprague Dawley (SD) rats and aggregation of washed platelets measured using light transmission aggregometry. Intracellular calcium ion concentration ([Ca^2+^]_*i*_) was measured using Fura-2/AM while ATP release evaluated by luminometer. Activation of integrin α_IIb_β_3_ analyzed by flow cytometry and clot retraction. Furthermore, we studied the signaling pathways mediated by *R. acetosa* extract by western blot analysis.

**Results:**

*R. acetosa* extract markedly inhibited collagen-induced platelet aggregation and ATP release in a dose-dependent manner. It also suppressed [Ca^2+^]_*i*_ mobilization, integrin α_IIb_β_3_ activation and clot retraction. The extract significantly attenuated phosphorylation of the MAPK pathway (i.e., ERK1/2, JNK), MKK4, PI3K/Akt, and Src family kinase.

**Conclusion:**

Taken together, this data suggests that *R. acetosa* extract exhibits anti-platelet activity via modulating MAPK, PI3K/Akt pathways, and integrin α_IIb_β_3_-mediated inside-out and outside-in signaling, and it may protect against the development of platelet-related cardiovascular diseases.

## Background

World Health Organization (WHO) disclosed (EURO/03/06) that cardiovascular disease (CVD) reveal the highest mortality among all diseases in western world. WHO has also stated that CVD accounted for 30% of all the deaths that occurred in 2005. In Europe, CVD remains the primary cause of death accounting for 42% of mortalities in men and 52% of deaths in women [1–3]. Coronary heart disease alone caused almost one in every seven deaths and heart failure caused one in nine deaths in the United States in 2013 [4]. Atherosclerotic plaque disruption and thrombogenic substrate exposure initiate platelet activation and aggregation, triggering coagulation cascade which lead to thrombus formation. Acute myocardial infarction and sudden death are the main clinical manifestations of atherosclerosis [5, 6]. For the last decades, antiplatelet drugs have been developed to prevent cardiovascular disease. However, these drugs have serious side effects; in particular, the side effects of aspirin are gastric ulcers and bleeding, and clopidogrel sometimes results in aplastic anemia and thrombocytopenic purpura [7]. Beside treatment of cardiovascular risk factors and use of antithrombotic agents there is considerable interest in traditional remedies and use of natural food products in prevention of CVD [8–12].

**Fig. 1:**
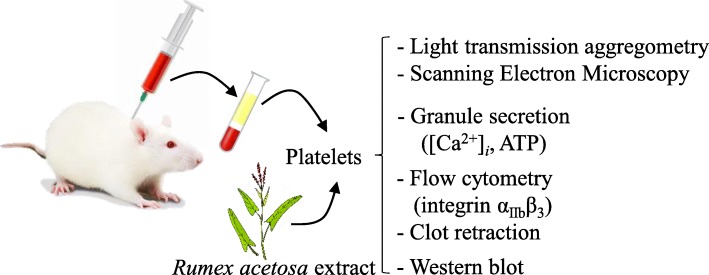
Study scheme to investigate in vitro effects of *R. acetosa* extract in rat platelets

In our effort to discover complementary materials, we found *Rumex acetosa* L, a natural product known to have ethnomedicinal properties. Plants were vital much before the human civilization. Specially, they have been used to intake as well as traditional medicine to improve health for years. The *Rumex* (dock) species have been used in medical treatment for many centuries owing to their astringent, spasmolytic, antithrombotic and cholagogic activity [13, 14]. *R. acetosa* is a perennial plant distributed in eastern Asia, Europe, and America [15] and the plant often called ‘Sorrel’, have been used within food technology and as phytotherapeutic materials in Korea and Japan [16]. The phytochemical components of *R. acetosa* extract have recently been identified to be monomeric flavan-3-ols (catechin, epicatechin, and epicatechin-3-O-gallate), A- and B-type procyanidins, and propelargonidins (15 dimers, 7 trimers, 2 tetramers) [17, 18]. This plant’s medicinal properties are related to its tannin content and are useful for the treatment of various ailments [19]. Previous studies reported that *R. acetosa* possessed antioxidant [19, 20], anti-hypertensive [21], antiviral, [22] and anticancer effects [23]. However, information on the antiplatelet effects of *R. acetosa* L extract remained to be discovered. Therefore, we investigated in vitro anti-platelet effect of *R. acetosa* extract and discovered underlying mechanism and signaling pathway in rat platelets **(**Fig. 1**).**

## Methods

### Chemicals and reagents

Thrombin and Collagen (Native collagen fibrils, type I, from equine tendons) were procured from Chrono-Log Co. (Havertown, PA, USA). ATP assay kit was acquired from Biomedical Research Service Center (Buffalo, NY, USA), while Fibrinogen Alexa Fluor® 488 conjugate was acquired from Molecular Probes (Eugene, OR, USA). Fura-2/AM and dimethyl sulfoxide (DMSO) were obtained from Sigma Chemical Co. (St. Louis, MO, USA). Antibodies against ERK (p44/42), phospho-ERK (p44/42), JNK, phospho-JNK, PI3K (p85/p55), phospho-PI3K (p85/p55), Akt (Ser473), phospho-Akt (Ser473), MKK4, phosphor-MKK4, Src (Tyr416), and phospho-Src (Tyr416) were obtained from Cell signaling (Beverly, MA, USA). Water was acquired from J. T. Baker (Phillipsburg, NJ, USA). All chemicals were of reagent grade.

### Procurement of plant material and extract preparation

Whole dried plant of *R. acetosa* was collected from Rural Development Administration (RDA) in Suwon city. Dr. Jeong-Hoon Lee and Dr. Seung-Eun Lee at RDA undertook the formal identification of plant materials on the basis of botanical characteristics. A voucher specimen of the plant material has been deposited in National Institute of Horticultural and Herbal Science (NIHHS), Eumseong, Republic of Korea, with Voucher ID (NIHHS 2012–026).

The powder (100 g) of *R. acetosa* plant was extracted with methanol in accelerated solvent extraction system (Dionex, USA) at 50 °C, and evaporated in rotary evaporator (N-1000, Eyela, Japan). Finally, crude extract was obtained and stored at − 30 °C for further use in experiments.

### Animals

Male Sprague Dawley (SD) rats (7 weeks old, ~ 240–250 g) were purchased from Orient Co. (Seoul, Korea). Rats were acclimatized for 1 week before the experiments and accommodated in an animal room with a 12/12-h light/dark cycle at a temperature and humidity of 22 ± 1 °C and 50 ± 10%, respectively. All experiments were carried out in accordance with the National Institutes of Health (NIH) guidelines and protocols approved by the Ethics Committee of the College of Veterinary Medicine, Kyungpook National University (Daegu, Korea); and later, rats were euthanized by an overdose of 5% isoflurane as previously described [24]. Isoflurane exposure was continued after 1 min of breathing stoppage, followed by cervical dislocation for confirmation of euthanasia.

### Platelet preparation

Preparation of washed platelets was conducted as previously described [25]. Briefly, whole blood was collected from rats via heart puncture and anticoagulated with ACD solution. Firstly, to obtain PRP, anticoagulated blood was centrifuged at 170×*g* for 7 min. Remaining RBC’s were removed by centrifuging the PRP at 120×*g* for 7 min. Subsequently, washed platelets were isolated by centrifuging the PRP at 350×*g* for 7 min. Platelets were resuspended in Tyrode’s buffer (137 mM NaCl, 12 mM NaHCO_3_, 5.5 mM glucose, 2 mM KCl, 1 mM MgCl_2_, and 0.3 mM NaHPO_4_, pH 7.4) and platelet concentration was adjusted at 3 × 10^8^ cells/mL. All the platelet preparation procedure was performed at room temperature (i.e., 23 ± 2 °C).

### Platelet aggregation assay and scanning electron microscopy analysis

Platelet aggregation assay was performed as previously described [26]. Aggregation was assessed by light transmission in an aggregometer (Chronolog, Havertown, PA, USA). Briefly, washed platelets were pre-incubated either with vehicle or different concentration of *R. acetosa* extract for 2 min at 37 °C, and then aggregation was induced with collagen for 5 min under continuous stirring condition. The vehicle concentration was held at less than 0.1%.

The scanning electron microscopy (SEM) analysis was performed using a Field Emission Scanning Electron Microscope (SU8220, Hitachi, Japan). After the termination of platelet aggregation, the washed platelets were fixed in 0.5% paraformaldehyde and Osmium tetroxide, dehydrated by ascending concentrations of ethanol, and freeze-dried and analyzed by the SEM.

### [Ca^2+^]_*i*_ measurement

The intracellular calcium mobilization ([Ca^2+^]_*i*_) was assessed with Fura-2/AM [27], and Fura-2 fluorescence in the cytosol was quantified with the spectro-fluorometer as previously described by Schaeffer and Blaustein [28] using the following formula: [Ca^2+^]_*i*_ 224 nM × (*F* − *F*_*min*_)/(*F*_*max*_ − *F*), where 224 nM is the dissociation constant of the Fura-2-Ca^2+^complex, and *F*_*min*_ and *F*_*max*_ represent the fluorescence intensity levels at very low and very high Ca^2+^ concentrations, respectively. Here, *F*_*min*_ and *F*_*max*_ is the fluorescence intensity of Fura-2-Ca^2+^complex measured at 510 nm when platelet suspension treated with 20 mM Tris/3 mM of EGTA and 1 mM of CaCl_2_ solubilized with Triton X-100 (0.1%), respectively; while *F* denotes the intensity when suspension is treated with collagen in presence or absence of *R. acetosa* L extract along 1 mM CaCl_2_.

### ATP release assay

Platelets were pre-incubated either with vehicle or different concentration of *R. acetosa* extract in the presence of 1 mM CaCl_2_ at 37 °C for 2 min prior to stimulation with collagen (2.5 μg/mL) for 5 min under continuous stirring condition. Reaction was stopped and suspension was centrifuged at high speed to collect supernatant. ATP concentration was assessed in luminometer (GloMax20/20; Promega, Madison, USA) using an ATP assay kit according to manufacturer’s protocol.

### Measurement of fibrinogen binding to integrin α_IIb_β_3_

Fibrinogen Alexa Fluor® 488 conjugate binding to integrin α_IIb_β_3_ on platelets was assessed by flow cytometry as previously described [29]. Briefly, washed platelets were pre-incubated either with vehicle or different concentrations of *R. acetosa* extract along with 0.2 mM CaCl_2_ for 2 min. The platelets were stimulated with collagen for 5 min, following incubation with fibrinogen Alexa Fluor® 488 (20 μg/mL) for 5 min at room temperature, and then fixed with 0.5% paraformaldehyde for 30 min at 4 °C. Alexa Fluor 488-fibrinogen binding to integrin α_IIb_β_3_ on platelets was quantified by flow cytometry using FACS Aria™ III flow cytometer® (BD Biosciences, San Jose, CA, USA) while data were analyzed using CellQuest software (BD Immunocytometry Systems, San Jose, CA, USA).

### Clot retraction

The in vitro effect of *R. acetosa* on outside-in signaling through integrin activation was assessed by measuring clot retraction as previously described [29]. PRP (250 μL) was incubated with vehicle, *R. acetosa* extract or Y-27632 (Rock inhibitor) for 2 min, following addition of RBC’s (5 μL) and Tyrode’s buffer to raise the volume up to 1 mL. Clot retraction was initiated by addition of thrombin (1 U/mL) and observed for 90 min at room temperature. Finally, clot weight was measured to assess clot retraction.

### Immunoblotting

Platelets were pre-incubated either with vehicle or different concentration of *R. acetosa* extract in the presence of 1 mM CaCl_2_ at 37 °C for 2 min prior to stimulation with collagen (2.5 μg/mL) for 5 min under continuous stirring condition. Reacting was terminated by adding lysis buffer [0.125 M Tris–HCl, pH 6.8; 2% SDS, 2% β-mercaptoethanol, 20% glycerol, 0.02% bromophenol blue, 1 μg/mL phenyl methyl sulfonyl fluoride (PMSF), 2 μg/mL aprotinin, 1 μg/mL leupeptin, and 1 μg/mL pepstatin A]. Proteins were quantified by BCA assay (PRO-MEASURE; iNtRON Biotechnology, Seoul, Korea) and total cell proteins (35 μg) from the lysates were segregated on 10% SDS-PAGE followed by transferring to polyvinylidene difluoride (PVDF) membranes. The membranes were blocked in 5% skim milk and then probed with primary and secondary antibodies accordingly in 5% BSA solution. Finally, antibody binding was pictured by enhanced chemiluminescence (iNtRON Biotechnology, Seoul, Korea).

### Statistical analysis

To assess statistical significance among observed differences, the obtained data were analyzed by one–way analysis of variance (ANOVA) followed by post-hoc Dunnett’s test (SAS Institute Inc., Cary, NC, USA). The given data are presented as the mean ± standard deviation (SD). *P*-values of 0.05 or less were considered statistically significant.

## Results

### Effect of *R. acetosa* extract on collagen-induced platelet aggregation

**Fig. 2:**
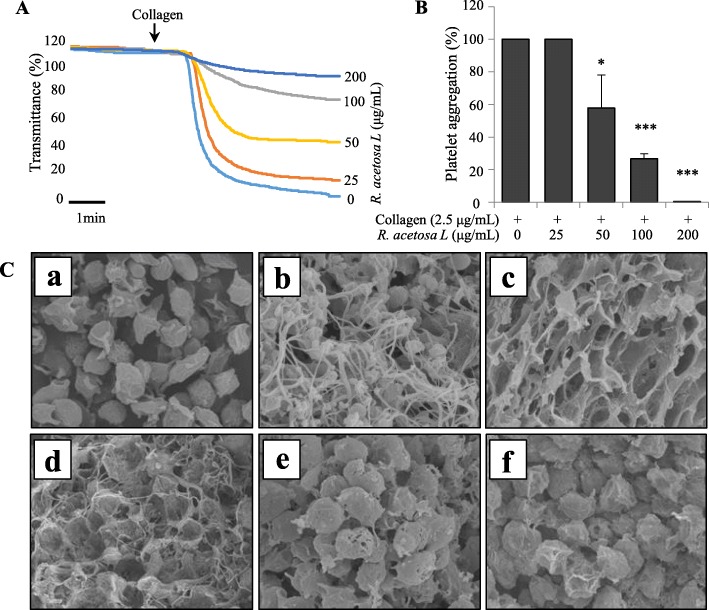
The inhibitory effect of R. acetosa extract on collagen-induced platelet aggregation. Platelets were pre-incubated with or without *R. acetosa* extract (25–200 μg/mL) in the presence of 1 mM CaCl_2_ for 2 min at 37 °C with stirring and stimulated with collagen (2.5 μg/mL) for 5 min (**a-c**). After the aggregation reaction was terminated, platelet aggregation was quantified and expressed as percentage. **c** Representative scanning electron microscopy images of platelets treated with various concentrations of extract or vehicle [Resting state (**a**), vehicle (**b**), *R. acetosa* extract 25 μg/mL (**c**), 50 μg/mL (**d**), 100 μg/mL (**e**), or 200 μg/mL (**f**)]. Each graph shows the mean ± SD of at least four independent experiments. *P < 0.05 and ***P < 0.001 compared to the agonist control

Our result showed that *R. acetosa* extract strongly inhibited platelet aggregation induced by collagen in dose dependent manner **(**Fig. 2a-b**)**. Platelet activation causes granule secretion, shape change and fibrin formation ultimately leading to platelet aggregation. We confirmed the effect of *R. acetosa* L extract on collagen-induced platelet shape change from inactivated to activated state of platelets under electron microscope and found dose dependent inhibition of platelet activation and shape change compared with vehicle treated platelets **(**Fig. 2c**)**.

### *R. acetosa* extract markedly reduced [Ca^2+^]_i_ mobilization and granule secretion

**Fig. 3:**
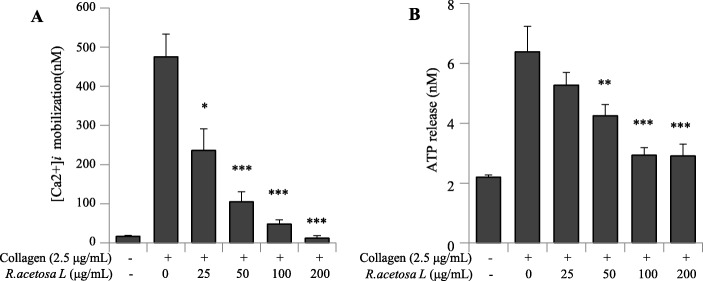
The inhibitory effect of R. acetosa extract on [Ca2+]i elevation and granule secretion. **a** Washed platelets were incubated with a calcium fluorophore (5 μM, Fura-2/AM), following treatment with different concentrations of *R. acetosa* extract and stimulated with collagen. **b** Platelets were pre-incubated with or without *R. acetosa* extract (25–200 μg/mL) in the presence of 1 mM CaCl_2_ for 2 min at 37 °C with stirring and stimulated with collagen for 5 min. Reaction was stopped and ATP release assay was carried out. The results are presented as the mean ± SD of at least four independent experiments. *P < 0.05, **P < 0.01 and ***P < 0.001 versus control

We found that pretreatment of platelets with *R. acetosa* extract markedly reduced the elevation in [Ca^2+^]_*i*_ in collagen stimulated platelets in a dose-dependent manner **(**Fig. 3a**)**.

Also, collagen strongly increased ATP release from dense granules in vehicle treated platelets by 3-fold in comparison with resting platelets. Our results show that platelets pretreated with *R. acetosa* extract significantly abridged ATP release in a dose-dependent manner **(**Fig. 3b**)**.

### Inhibitory effect of *R. acetosa* extract on inside-out and outside-in signaling

**Fig. 4:**
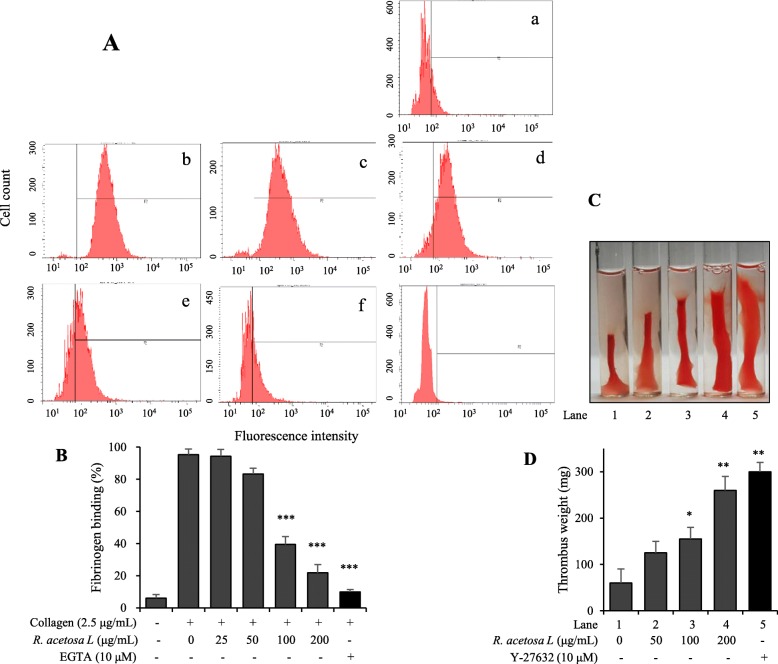
The inhibitory effect of R. acetosa extract on inside-out and outside-in signaling. Platelets were pre-incubated with or without *R. acetosa* extract (25–200 μg/mL) in the presence of 0.2 mM CaCl_2_ for 2 min at 37 °C with stirring and stimulated with collagen for 15 min at 37 °C. (A) Representative FACS analysis results of five independent trials [Resting (**a**), Vehicle (**b**), *R. acetosa* extract 25 μg/mL (**c**), 50 μg/mL (**d**), 100 μg/mL (**e**), 200 μg/mL (**f**) and, EGTA 10 μM (**g**)]. **b** Bar graph summarizing the inhibitory effect of *R. acetosa* extract on fibrinogen binding. **c-d** Thrombin (1 U/mL) was used to initiate clot retraction in presence or absence of *R. acetosa* (50–200 μg/mL) or Y-27632 (10 μM). Clot retraction was observed for 90 min and representative images of clot retraction at 1 h (**c**), while bar graph (**d**) showing inhibitory effects of *R. acetosa* extract on clot retraction. *P < 0.05, **P < 0.01 and ***P < 0.001 versus control

We found that *R. acetosa* extract reduced affinity of fibrinogen binding to integrin α_IIb_β_3_**(**Fig. 4a-b**)** and clot retraction via Rho kinase inhibition in a dose-dependent manner **(**Fig. 4c-d**)**.

### Effect of *R. acetosa* extract on MAPK, PI3K/Akt and Src phosphorylation

**Fig. 5:**
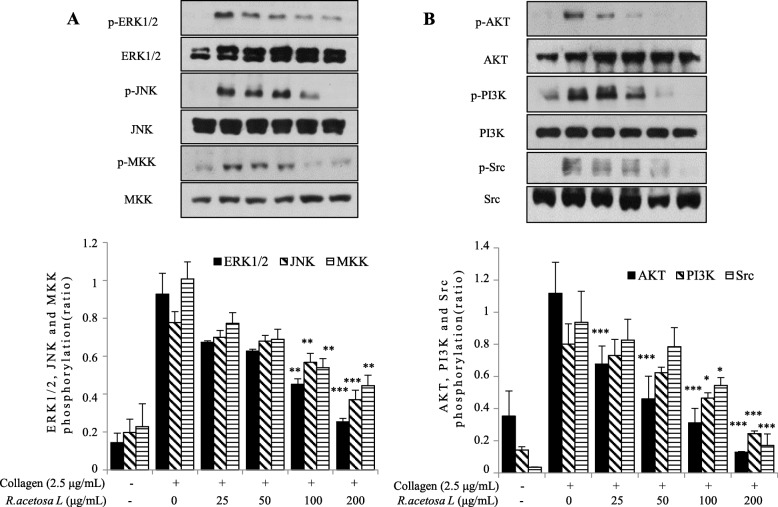
*R. acetosa* extract attenuated the phosphorylation of MAPK, PI3K/Akt, and Src family *kinase*. After the platelet aggregation reaction, platelet proteins were then extracted and specific antibodies were used to measure the levels of total and phosphorylated ERK, JNK, MKK4, PI3K/Akt, and Src family kinase. All immunoblots were carried out in at least three independent experiments. **P < 0.01 and ***P < 0.001 compared to the agonist-treated group

To explore the underlying mechanism, we further studied the phosphorylation of downstream signaling proteins including MAPK’s and MKK4. Our result shown that *R. acetosa* extract reduced the phosphorylation of ERK1/2 and JNK. *R. acetosa* extract also inhibited the phosphorylation of MKK4 which is an upstream signaling molecule of JNK **(**Fig. 5a**)**.

Our result also revealed that *R. acetosa* extract markedly inhibited collagen-induced PI3K/Akt signaling in a dose-dependent manner. In addition, phosphorylation of Src family kinases plays a role in GPVI-mediated platelet activation and we found that *R. acetosa* extract significantly reduced the activation of Src family kinase **(**Fig. 5b**)**.

## Discussion

Cardiovascular diseases such as atherosclerosis, thrombosis, and myocardial infarction are the major causes of mortality in the modern world. Platelets play critical roles in hemostasis, thrombosis, immunity, and inflammation. At the site of vascular injury, platelets are activated by agonists such as collagen, adenosine diphosphate (ADP), and thrombin. These agonists initiate signal transduction through their specific receptors leading to platelet morphologic changes, granule secretion, and aggregation [30]. However, aberrant or over-activation of platelet formation induces a platelet plug and thrombus formation, which can lead to serious ailments such as atherosclerosis. Therefore, the development of anti-platelet agents is a basic goal in cardiovascular research [31].

In the present study, we explored whether *R. acetosa* extract inhibits collagen-stimulated platelet activation and our results showed that *R. acetosa* extract markedly inhibited collagen-stimulated platelet aggregation in a dose-dependent manner. To demonstrate the inhibitory mechanism of *R. acetosa* extract, we further examined downstream signaling components such as intracellular calcium mobilization, granule secretion, integrin signaling, and various proteins phosphorylation. Cytosolic calcium level is known to play a critical role in platelet activation. Increasing calcium levels activates several signaling pathways involved in actin-myosin interaction, protein kinase c (PKC), calmodulin, and calcium-dependent proteases [32]. Our results show that *R. acetosa* extract strongly inhibited the intracellular mobilization of calcium. Intracellular Ca^2+^ mobilization is also essential for α- and δ-granule secretion [33]. Granule secretion improves platelet activation and recruitment of circulating platelets into injured blood vessels. It is also important for thrombus formation. In this study, we found that treatment with *R. acetosa* extract decreased ATP release from dense granules in collagen-stimulated platelets. Platelets express integrins such as α_IIb_β_3_ (fibrinogen receptor), α_2_β_1_ (collagen receptor), and α_V_β_1_ (fibronectin receptor). These integrin’s regulate signal transduction by various mechanisms. When a specific ligand binds to integrin α_IIb_β_3_, the fibrinogen receptor changes its conformational structure which enhances affinity to bind with fibrinogen following platelet adhesion and clot retraction [34]. Our data suggests that pretreatment with *R. acetosa* extract dose-dependently blocked fibrinogen binding to integrin α_IIb_β_3_ and clot retraction.

**Fig. 6:**
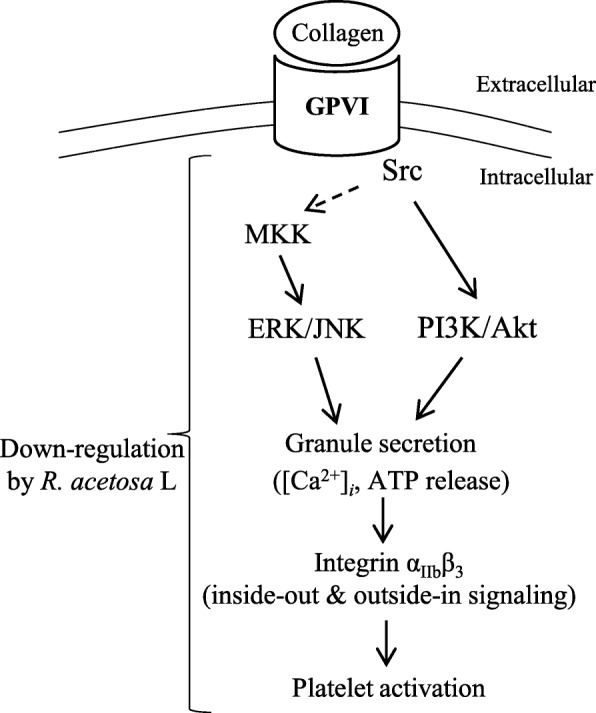
Summary of effects of *R. acetosa* extract on platelet intracellular signaling

Mitogen-activated protein kinases (MAPKs), including ERK1/2 and JNK1, exist in platelets and mediate proliferation, migration, and apoptosis. MAPKs are phosphorylated by several agonists, such as collagen, ADP, and thrombin, and are important for “outside-in” as well as “inside-out signaling” [35]. The PI3K/Akt signaling pathway also is critical for platelet activation and aggregation. Further, PI3Ks are necessary for the tyrosine phosphorylation-based signaling pathways initiated by GPVI or α_IIb_β_3_ [36]. The results of our study showed that *R. acetosa* extract inhibited phosphorylation of ERK1/2, JNK, and MKK4. Moreover, the PI3K-Akt pathway was also blocked by *R. acetosa* extract. Specific ligand binding to GPVI, the immunoreceptor tyrosine-based activation motif (ITAM) within the FcRγ cytoplasmic domain, is a tyrosine moiety phosphorylated by Src family kinases (including Fyn and Lyn). Phosphorylation of Src family kinases is important for GPVI-mediated platelet activation [36]. Our data showed that SFK phosphorylation induced by collagen was significantly decreased by treatment with *R. acetosa* extract. Figure 6 showing a graphical summary of effects of *R. acetosa* extract on platelet intracellular signaling.

Previous reports have suggested that methanolic extract of *R. acetosa* contained several pharmacological compounds such as catechin, epicatechin and epigallocatechin-3-O-gallate [19, 37], while these compounds have been known for their antiplatelet activities [38]. Therefore, antiplatelet effects observed in present study could be attributed to catechin and epicatechin contained in *R. acetosa* extract. We acknowledge that there are some limitations i.e., study could explore in vitro antiplatelet properties of extract in collagen-stimulated rat platelets. Future studies may be planned to discover in vivo antiplatelet aspects and unravel other pathways involved in its antithrombotic mechanism.

## Conclusion

We conclude that *R. acetosa* extract has potent antiplatelet effects and good candidate in the new era of ethnomedicine against cardiovascular diseases, including atherosclerosis, ischemic stroke, and myocardial infarction. Future studies could explore further in vivo effects of the extract and validate its pharmacological compounds in animals and humans as potential antithrombotic agents.

## Data Availability

The dataset generated during the present study is available upon reasonable request to the author (Prof. Man Hee Rhee).

## Abbreviations

ADP: Adenosine diphosphate
Akt: Protein kinase B
DMSO: Dimethyl sulfoxide
ERK: Extracellular signal-regulated kinase
GPVI: Glycoprotein VI
JNK: c-Jun N-terminal kinase
MAPK: Mitogen-activated protein kinase
MKK4: Mitogen-activated kinase kinase 4
PI3K: Phosphatidylinositol 3-kinases
PRP: Platelet-rich plasma
SFK: Src family kinase
